# Reducing antibiotic duration for acute otitis media: clinician, administrator, and parental insights to inform implementation of system-level interventions

**DOI:** 10.1017/ash.2024.469

**Published:** 2025-01-06

**Authors:** Deborah J. Rinehart, Aiden Gilbert, Sonja O’Leary, Sophie E. Katz, Holly M. Frost

**Affiliations:** 1 Center for Health Systems Research, Office of Research, Denver Health and Hospital Authority, Denver, CO, USA; 2 Division of General Internal Medicine, School of Medicine, University of Colorado Anschutz Medical Campus, Aurora, CO, USA; 3 Department of General Pediatrics, Denver Health Medical Center, Denver, CO, USA; 4 Department of General Pediatrics, University of Colorado School of Medicine, Aurora, CO, USA; 5 Division of Pediatric Infectious Diseases, Vanderbilt University Medical Center, Nashville, TN, USA

## Abstract

**Objective:**

This qualitative study aimed to understand facilitators and barriers to implementation of interventions to improve guideline-concordant antibiotic duration prescribing for pediatric acute otitis media (AOM).

**Design:**

Clinicians and clinic administrators participated in semi-structured qualitative interviews, and parents of children 2 years of age or older with a recent diagnosis of AOM participated in focus groups. The Practical Robust Implementation and Sustainability Model (PRISM) guided the study. Interviews were analyzed using the Rapid Assessment Process.

**Setting:**

Denver Health and Hospital Authority (Denver, CO) led the study. Recruitment occurred at Vanderbilt University Medical Center (Nashville, TN) and Washington University in St. Louis Medical Center (St. Louis, MO).

**Participants:**

Purposeful sampling was used to recruit clinicians and administrators for qualitative interviews. Convenience sampling was used to recruit parents for focus groups.

**Results:**

Thirty-one participants (15 clinicians, 4 administrators, and 12 parents) engaged in interviews and focus groups. Factors influencing antibiotic prescribing included patient history, years of practice, familiarity with the patient, concerns with patient medication adherence, and practice type. Clinicians endorsed electronic health record modifications and clinician prescribing feedback as methods to improve patient care and reduce the durations of prescribed antibiotics. Suggestions for intervention optimization and education needs were also obtained.

**Conclusions:**

Findings suggest that clinicians and administrators support reducing prescribed antibiotic durations for AOM and are receptive to the proposed interventions. More education is needed to increase parent awareness about antibiotic stewardship and AOM treatment options.

**Clinical trials identifier:**

RELAX: Reducing Length of Antibiotics for Children with Ear Infections (RELAX), NCT05608993, https://clinicaltrials.gov/study/NCT05608993.

## Introduction

Acute otitis media (AOM) is the leading reason children in the United States are prescribed antibiotics.^1,2^ Nearly 85% of children improve without antibiotics,^3^ and short (5 days) and long (10 days) antibiotic durations are similarly effective for most children 2 years and older.^4^ However, longer courses pose a higher risk of adverse drug events and may confer a greater risk of subsequent antimicrobial resistance.^5,6^ Thus, the American Academy of Pediatrics (AAP) recommends short courses (5–7 days) for most children 2 years of age and older,^2^ yet over 90% of children are prescribed 10-day durations.^7,8^

We previously reported that in a single center, bundled antibiotic stewardship interventions for AOM reduced the use of long antibiotic durations without increasing treatment failure or recurrence.^9,10^ An intervention that included individualized audit and feedback for clinicians, comparing their prescribing to their peers, was more effective than one without. However, audit and feedback can require significant resources and may be challenging to implement in some settings.^11^ Our current study, Reducing Length of Antibiotics for Children with Ear Infections (The RELAX Trial/NCT05608993), uses a multi-center cluster randomized clinical trial to compare the effectiveness of a low-intensity intervention (education and electronic health record (EHR) changes) to a high-intensity intervention (adding individualized audit and feedback) in reducing the use of long antibiotic durations for children 2 years of age and older with uncomplicated AOM.^12^

This formative qualitative study aims to understand multilevel factors affecting antibiotic prescribing for AOM and optimal strategies for the implementation of 2 stewardship interventions (EHR changes and individualized prescribing feedback) focused on the duration of antibiotics for AOM in new settings.

## Methods

### Study design

The study team conducted semi-structured qualitative interviews with clinicians and clinic administrators from 2 large, regionally separated healthcare systems. Focus groups were conducted with parents of children who had previously been diagnosed as having AOM. The Practical Robust Implementation and Sustainability Model (PRISM) guided the study.^13,14^ PRISM is a dissemination and implementation science framework that specifies multilevel factors important in informing intervention implementation. This manuscript was written in accordance with the Consolidated Criteria for Reporting Qualitative Studies (COREQ) checklist,^15^ and the Colorado Multiple Institutional Review Board approved all study procedures.

### Setting and participant recruitment

Denver Health and Hospital Authority (Denver, CO) led the study. Participant recruitment occurred at 2 healthcare systems: Vanderbilt University Medical Center (Nashville, TN) and Washington University in St. Louis Medical Center (St. Louis, MO). Between April and June 2023, study staff at the 2 healthcare systems used purposeful sampling to recruit clinicians and administrators (management perspective) for qualitative interviews attempting to include at least 2 administrators from each healthcare system and representation from rural, urban, and suburban settings as well as primary care, urgent care, and retail clinics. After completing these interviews, convenience sampling was used to recruit parents of children 2 years of age or older with a recent diagnosis of AOM for focus groups. Participants were recruited via email and phone. All participants completed an informed consent and a demographic survey in REDCap.^16,17^

### Study procedures

Interviews ranged from 30 to 60 minutes and were conducted virtually and audio-recorded using WebEx (Cisco, San Jose, CA). One of 2 female master’s (A.G.) or PhD-level (D.R.) researchers with training and experience in qualitative methods conducted the interviews. Focus groups were also conducted virtually using WebEx and were moderated by one of the qualitative researchers and a study clinician. Focus groups lasted between 60 and 90 minutes. Participants received a $50 gift card for their participation.

**Figure 1.**
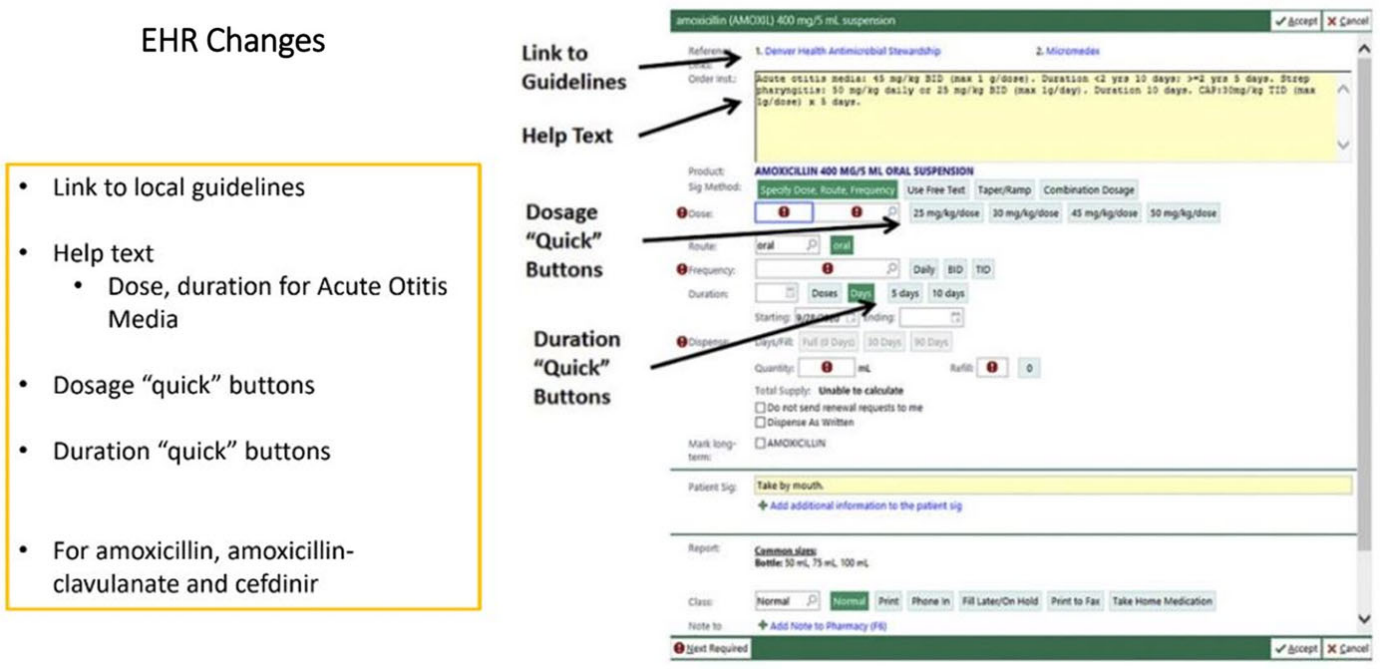
Interview intervention materials—electronic health record changes.

**Figure 2.**
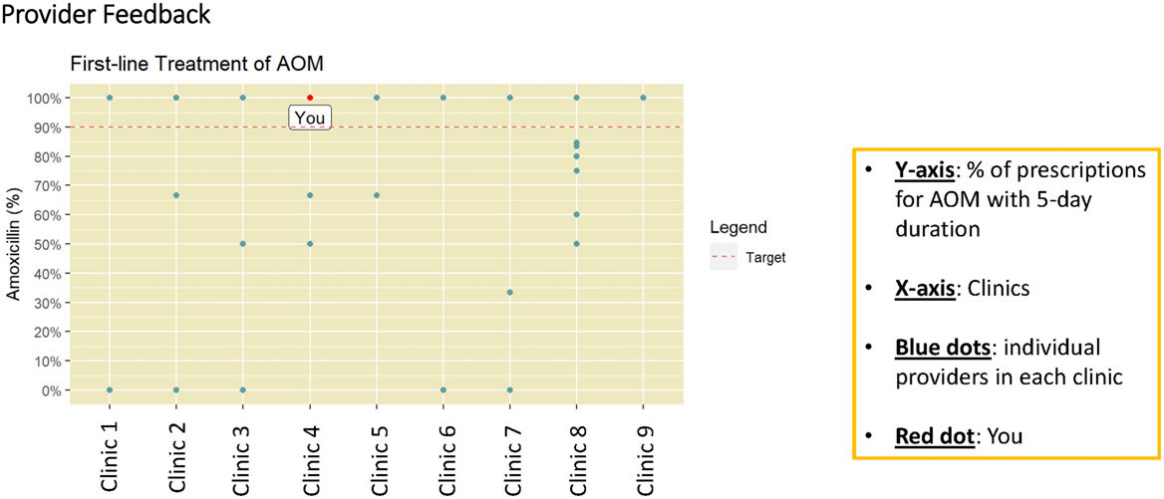
Interview intervention materials—provider feedback.

The study team developed 2 interview guides, 1 for clinicians and 1 for administrators (Supplement 1). The clinician guide focused on understanding individuals’ clinical practice behaviors, perception of other clinicians’ practices, and the clinic environment. The administrator guide focused on intervention implications at the organizational level. Both guides included open-ended questions to understand the following contextual domains: (1) factors related to the decision to prescribe antibiotics and factors that may facilitate or impede prescribing for short durations (5-day duration for uncomplicated AOM in children ≥ 2 years of age); (2) factors facilitating or impeding implementation of each intervention component (EHR modifications and prescribing feedback), as well as parental and clinician educational needs; and (3) understanding organization culture and openness regarding intervention implementation. Figures 1 and 2 are the graphics that were presented during the interviews to describe the 2 study interventions. Interviews were conducted until thematic saturation of the above domains was reached.

A focus group guide was also developed (Supplement 1) to understand parent experiences with AOM and treatment, individual and contextual factors influencing parental perception regarding using short antibiotic durations, and input on education needs for parents and study educational pamphlets.

### Data analysis

Interview data were analyzed using the Rapid Assessment Process^18^ (Supplement 2). Themes were summarized by key domains. The research team met regularly with each other and the broader study team (inclusive of clinicians at each site) to identify emergent themes, assess for saturation, and obtain feedback. Focus groups were recorded, and moderators compiled a summary document following the meeting. The team met to discuss themes among the 2 groups.

## Results

### Characteristics of study participants

The study team conducted 19 individual clinician/administrator interviews and 2 parent focus groups (a total of 12 participants; 7 in group 1 and 5 in group 2); demographics are presented in Table 1. Most clinicians and administrators (84%) had over 10 years of experience. All focus group participants had at least 1 child with recurrent AOM, many (86%) of whom had prior tympanostomy tubes. Themes for interviews and focus groups are organized below by the PRISM Domains and the high-level themes and quotes for each domain can be found in Table 2.

**Table 1.** Participant characteristics—clinician and administrator interviews and caregiver focus groups

	Clinicians and administrators (N=19^b^)	Caregivers (N=12)
	N (%)	N (%)
Age		
18–29	0 (0)	1 (8)
30–39	4 (21)	5 (42)
40–49	7 (37)	6 (50)
50–59	5 (26)	0 (0)
60+	3 (16)	0 (0)
Gender		
Female	14 (74)	9 (75)
Male	5 (26)	3 (25)
Non-binary	0 (0)	0 (0)
Race^a^		
American Indian/Alaska Native	0 (0)	1 (8)
White	16 (84)	11 (92)
Native Hawaiian/Pacific Islander	0 (0)	0 (0)
Black/African American	0 (0)	0 (0)
Asian	3 (16)	1 (8)
Ethnicity		
Hispanic	1 (5)	0 (0)
Non-Hispanic	18 (95)	12 (100)

a Select all that apply.

b Clinical specialty was only asked of MD-level clinicians (n = 11); 10 were in pediatrics, and 1 was in family medicine.

Vanderbilt University Medical Center: n = 14 (10 clinicians/administrators and 4 caregivers).

Washington University at St. Louis: n = 17 (9 clinicians/administrators and 8 caregivers).

**Table 2.** Interview themes by PRISM domains

PRISM domain	Themes	Quotes
Multilevel characteristics of implementers and intended audience (factors influencing antibiotic prescribing and duration)	Individual level (Clinician) o Patient clinical presentation and history o Habits and years in practice o Connection/familiarity with patient/family o Concern with medication adherence (Parent) o Lacking information on treatment options and antibiotic side effects o Perceived need/expectation for antibiotics o Pain management concerns Organizational level o Practice setting (urgent care, primary care) o Consistency is important across providers and settings	Individual level—clinician “I think everyone is kind of stuck with what they’re comfortable with, most providers find it hard to change.” (Int. 009) “Hopefully all of us use the art of medicine as well as the science when deciding how long to prescribe which antibiotic to use based on child’s medical and social history…and vibes you are getting from parents.” (Int. 001) Individual Level – Parent “Once I decide to take her in, I expect to get treatment (antibiotics).” (FG #2) “I don’t recall that the pediatrician ever made me aware of the issues that this (antibiotics) may cause when she takes antibiotics so frequently…so that’s something that I wish was made clear to me, because it may have changed my perspective on how to treat and the treatment we choose for my child.” (FG #1) Organizational Level “We all know the challenges with antibiotic over-use…I think with urgent cares, it is (antibiotics) the quick answer to get people in and out.” (Int. 010)
Multilevel perspectives on intervention components	Clinician/administrator perspectives • Electronic Health Record (EHR) o Do not add time and minimize added clicks o Allow flexibility so a provider can override o Provide EHR efficiencies to save as a favorite and replace old templates o Include staff and strong communication during the rollout • Audit Feedback o Ensure feedback is anonymous and focus on improving rather than shaming o Clarity on outcome measure and compare like settings/populations o Send quarterly from a familiar person/expert • Education o Provide updated studies/data/guidelines for shorter prescribing durations o Parental information should be available in various formats o Educate parents on when to bring child in for a visit Parent perspectives • More education on antibiotic durations/side effects, monitoring symptoms, risks associated with treatment options • Most caregivers open to short durations with more information from trusted provider on treatment and risks • Desire for shared decision-making and data/studies on antibiotic treatment options • Share information during visit, handouts, and after-visit summaries	Clinician/admin perspectives “I never like changes to the EHR…changes muscle memory of where we click, always a little bit disorienting but in this specific scenario… there is not an extra thing that I have to click.” (Int. 17) “This would definitely drive people to change their practice. Nobody likes to be an outlier. I think if you saw those metrics, and feedback on the actual prescriptions, that would be great.” (Int. 005) Parent perspectives “I never want to leave the doctor’s office feeling like I need to Google something, because you never know if that is trustworthy or not. The reason I take my child to this pediatrician is because I have trust in them, so if he did not have time to explain, a pre-printed flyer that parents could take home and not take up the clinician’s time would be really good to have.” (FG #1) “This information is not readily available, and it is important for parents and caregivers to know because it may possibly change the treatment for the child. It is definitely important information.” (FG #1) “The shortest course (of antibiotics) would be ideal.” (FG #2) “I would like to see data, a journal article, something to crunch the numbers with all the information and make the best choice for your child.” (FG #1)
Implementation and sustainability infrastructure (organizational factors related to intervention implementation)	• Organizational support and openness to change when backed by evidence • Challenges if clinicians feel coerced to change practices (still need to provide an opportunity to make a decision) • Importance of unifying practices and messaging to caregivers within individual clinics and across the healthcare system • Need to ensure infrastructure is in place to support practices (if don’t prescribe—options for patients to get antibiotics if needed without a new visit, to communicate with patient)	

### Factors influencing antibiotic prescribing and duration

#### Individual level: clinician and parent perspective

At the individual level, *clinician-level themes* related to antibiotic prescribing for AOM included patient clinical presentation and history, clinician years in practice, familiarity with the family, and perceptions of patient medication adherence. Primary factors for prescribing decisions were the child’s age, clinical severity (ear fluid/swelling, pain, fever), history of AOM infections, other health conditions, medication allergies, and past treatment failures. Clinicians typically prescribed amoxicillin with most (8, 57%) prescribing 7–10-day durations, and few (4, 29%) prescribing 5-day durations. Increasing years of experience was identified as a factor that would decrease the likelihood of modifying prescribing habits.

Familiarity between clinician and family also influenced prescribing. Strong rapport and trust increased the likelihood of using short durations or delayed prescribing. Concerns about medication adherence, such as completing the full duration, impacted management (eg, prescribing 10 days to ensure at least 7 days of treatment, or 7 days hoping for at least 5 days). Some clinicians expressed concern about the adequacy of a 5-day prescription.

*Parent-level themes* related to antibiotic prescribing included a lack of information on treatment options and side effects and the belief that clinical improvement requires antibiotics. Parents generally desired more information about AOM treatment options. Historically, the perception was that clinicians almost always prescribed a 10-day antibiotic course, and most parents were unaware of the 5-day option. Many were open to shorter durations and trusted their clinician’s judgment. Some noted that clinicians rarely discussed the side effects of antibiotics. Similarly, parents indicated that clinicians rarely or never discussed shorter-duration options and felt they needed more information.

Parents often waited until their child’s pain persisted for several days before seeking clinical evaluation. They typically expected antibiotics and effective pain management as part of the evaluation. They were concerned about symptom worsening, needing follow-up appointments, or permanent hearing loss if antibiotics were not prescribed. Additionally, parents reported inadequate information on pain management, with clinicians offering only vague advice to treat pain “as needed,” leading many to seek more guidance elsewhere.

#### Organizational level

Clinicians and administrators identified 3 *organizational-level themes* impacting prescribing: practice setting workflows, trust associated with care continuity, and prescribing culture. In primary care, infrastructure including MyChart (EPIC, Verona, WI) use and available administrative staff enables follow-up via electronic message or phone, promoting flexibility in prescribing and reducing return visits and co-pays. Clinicians emphasized that differences in continuity between primary care and urgent care affect clinician/family rapport and prescribing practices. In primary care, clinicians establish relationships with families through continuity heightening parental trust in the clinician’s management as well as clinician trust related to the parent’s history of medication adherence. In contrast, in urgent care settings, many patients present after-hours, with more acute symptoms, and without a prior relationship with the clinician. Clinicians usually see patients only once for a brief visit and lack follow-up mechanisms for concerns that may arise. Lack of continuity, perceived parental expectations, and unfamiliarity with the child’s medical history drive urgent care clinicians to prescribe antibiotics or use longer durations.

Regardless of the setting, the most prominent organizational-level theme identified was the importance of consistency in prescribing practices. Most agreed that while prescribing practices were generally consistent within their practice, variations (eg, differing antibiotic durations, recommending watchful waiting) undermine patient trust. Both clinicians and administrators stressed the need for a unified approach, particularly within primary care but also across different settings.

### *Perspectives on intervention components (see* Figures 1 and 2 *for intervention components)*

#### Individual level: clinicians and administrators

##### Electronic health record

Feedback on proposed EHR changes to support shorter antibiotic durations was generally positive. Key themes included minimizing additional clicks, allowing clinicians’ flexibility to override settings, replacing old templates, and strong collaboration and communication during rollout. Time efficiency is crucial for uptake, and prefilled directions for medication (dosage and duration quick buttons) were well-received. Some requested a 7-day duration button in addition to the existing 5- and 10-day options. Additionally, accuracy in dosage amounts was a concern for a few. Clinician autonomy was emphasized as important.

Participants noted the importance of clear communication with staff to ensure buy-in and understanding. They stressed replacing existing templates/favorites in the EHR to ensure clinicians adopt the new settings. Although most felt the changes would facilitate shorter antibiotic prescribing, some administrators were concerned about the length of time needed to make EHR changes at their organization.

##### Audit and feedback

Themes regarding individual clinician audit and feedback with peer comparison included keeping feedback anonymous and positive, clarifying the prescribing metric, and limiting comparisons to similar settings. Participants emphasized the importance of de-identified feedback to avoid targeting clinicians. Clear objectives and metrics were preferred, focusing on collective improvement rather than “individual shaming.” They suggested comparing similar patient populations and practices within similar settings, like urgent care clinics, rather than a mix of urgent and primary care settings. Quarterly feedback was favored, as higher-frequency reports might be ignored. Feedback from a familiar figure, such as a medical director, was recommended. Overall, most believed this approach would positively impact antibiotic stewardship and patient outcomes if done well.

##### Education

Clinicians and administrators highlighted the need for increased clinician familiarity with data supporting 5-day antibiotic durations. Almost all participants agreed on the necessity for more overall education on AOM and antibiotic stewardship, including AOM diagnosis, antibiotic selection and duration, vaccine impact, and watchful waiting. Clinicians felt that drivers of over-prescribing are often outside the pediatric settings (eg, in adult and urgent care settings). Many clinicians expressed a need for education on working with families with lower health literacy.

Clinicians also emphasized the importance of parental education on AOM, particularly monitoring symptoms at home and criteria for a healthcare visit. Specific needs for parent education, as expressed by clinicians, included information on viral AOM etiologies, self-resolution, watchful waiting rationale, antibiotic side effects, antibiotic duration, and information for high-risk patients. Administrators supported this education, especially for parents of children with recurrent AOM. For dissemination, preferred formats included physical flyers and EHR-generated after-visit summaries.

#### Individual level: parent perspective

##### Education

Themes from parents included a desire for more information on AOM and antibiotic use, shared decision-making, access to data and studies, and educational materials. Parents wanted information on the efficacy of short antibiotic durations, side effects, home care instructions, symptom monitoring, and follow-up indications. Most were open to short durations especially if informed by a trusted clinician and emphasized being part of the decision-making process. They preferred information during visits via handouts or after-visit summaries.

Parents reviewed a draft of a study parent education pamphlet and provided feedback for improvement. They appreciated clear information on the 5-day treatment, but some were confused by a graphic explaining why to delay antibiotics and recommended changes to improve clarity. Parents strongly disliked the use of the word “harm” when discussing antibiotics and suggested the language be tempered to terms like “side effects.”

#### Organization-level factors

Key themes regarding organizational culture and intervention implementation further highlighted the importance of sharing data supporting shorter antibiotic durations, the importance of clinician autonomy, the need to unify practices through strong communication, and ensuring supportive infrastructure. Clinicians and administrators felt their organizations would back the changes if supported by clinical evidence. They again emphasized the need for clinician autonomy and consistent messaging to maintain patient trust. Infrastructure support, including mechanisms for patients to schedule follow-up appointments, talk with a nurse, or have a prescription called if needed, should align with prescribing practices.

## Discussion

In this qualitative study, we provide valuable information from multiple perspectives to better understand factors influencing antibiotic prescribing behaviors for AOM and factors that should be considered when implementing antibiotic stewardship interventions. These formative findings highlight the importance of assessing contextual factors prior to intervention implementation and will be used to inform the implementation of 2 clinician-level stewardship interventions aimed at reducing antibiotic prescribing durations among children 2 years of age and older with uncomplicated AOM at 2 geographically diverse healthcare organizations.^12^

Beyond patient presentation, multiple factors were found to influence the duration of antibiotic prescribing. We noted that few clinicians opted for short antibiotic durations. There appeared to be a knowledge gap between current practice, AAP recommendations for AOM,^2^ and evidence for shorter durations.^4^ This underscores the need for better clinician education and improved dissemination of guidelines. Surprisingly, clinician concerns about nonadherence to the recommended duration led to prescribing of longer durations, despite evidence from previous studies indicating higher adherence with shorter durations.^19^ Utilization and preference for a 7-day treatment duration was a recurring theme in our interviews; however, a 7-day duration may be longer than necessary, particularly because many patients may not require antibiotic therapy. Most prior studies have compared 5- versus 10-day durations rather than 5- versus 7-day or 7- versus 10-day durations.^4^ This is likely due to the small absolute benefit from longer antibiotic durations and, consequently, the sizable patient sample that would be required to power such a study. Moreover, shorter treatment durations are regularly used for conditions considered more severe than AOM, such as community-acquired pneumonia.^20^ Integrating open discussions about 5- versus 7-day treatment durations for AOM into the interventions will be important.

We found that parents want to be included in their child’s treatment decisions. Many parents lacked knowledge about the risks and benefits of antibiotics and were unaware of alternatives like watchful waiting or shorter antibiotic durations. Misconceptions may lead parents to see antibiotics as harmless and to overstate the risks of not using them, such as fearing hearing loss from a single episode of AOM. Parents also viewed antibiotics as the best solution for their child’s discomfort. This perspective contrasts sharply with clinicians’ understanding; they often prescribe antibiotics assuming it meets parents’ expectations. However, there is no direct correlation between parental satisfaction and antibiotic prescription,^21,22^ revealing a significant communication gap.

Organizational factors likely play a vital role in reducing antibiotic prescribing and duration. Ensuring consistency in prescribing practices among clinicians and across clinics is important for building trust with parents, especially in practices where patients may see different clinicians. This consistency is also important across different settings, such as urgent and primary care. An organizational infrastructure that supports families managed with watchful waiting or shorter antibiotic durations is essential. Both parents and clinicians emphasized the need for an efficient, low-cost method for parents to communicate with a clinician after an appointment if needed.

Clinicians, administrators, and parents viewed the proposed interventions positively. Clinicians emphasized the need for education and quick access to clinical care guidelines. They also appreciated efforts to reduce the number of clicks in the EHR and streamline prescribing. Although clinicians felt that audit and feedback reports would improve care, they highly valued their privacy and wanted data to remain anonymous. Administrators stressed the importance of including support staff (nurses, medical assistants, and appointment staff) in intervention decisions and being aware of how proposed changes would impact workflow and parent satisfaction. Parents echoed the need for more education, emphasizing access to information after the visit. They recommended providing concrete instructions on pain management, when to start an antibiotic if watchful waiting was advised, and what to do if their child’s condition worsens or does not improve.

Strengths of this study include incorporating diverse viewpoints from parents, clinicians, and administrators across geographically separated health systems. The use of rapid analysis^18^ facilitated broad stakeholder participation in the analytic process and allowed for quick adaptation of interventions and implementation strategies. However, the study had some limitations. This was a small sample of participants who were interested in this topic and the viewpoints expressed by our participants may not be generalizable to all populations or health systems. We had an overrepresentation of parents with children experiencing recurrent AOM, who may have different knowledge and beliefs compared to those with fewer AOM episodes. To ensure full participation in the focus groups, we limited involvement to English-speaking individuals, and most participants were white and non-Hispanic. Notably, participants provided feedback on proposed interventions using mock-up materials rather than based on actual materials and their lived experiences using them in clinical settings.

In conclusion, this study highlights important insights into antibiotic prescribing practices for AOM and reveals multiple factors, beyond patient presentation, influencing these practices. These discoveries underline the necessity of improved clinician education, consistent messaging within healthcare organizations, strengthening clinician-parent communication, and involving parents more in treatment decisions to reduce unnecessary or overly long antibiotic prescriptions for children.

## Supporting information

Rinehart et al. supplementary material 1 Rinehart et al. supplementary material

Rinehart et al. supplementary material 2 Rinehart et al. supplementary material

## Supplementary material

The supplementary material for this article can be found at https://doi.org/10.1017/ash.2024.469.

## Research transparency and reproducibility

Study data is not available as it includes participant identifiers.
