# Supplementary material for: Reducing antibiotic duration for acute otitis media: clinician, administrator, and parental insights to inform implementation of system-level interventions
Source: Antimicrob Steward Healthc Epidemiol. 2025 Jan 6;5(1):e3. doi: 10.1017/ash.2024.469 (PMC11704944; doi:10.1017/ash.2024.469)
Supplement: Rinehart et al. supplementary material 1 — Rinehart et al. supplementary material [file S2732494X24004698sup001.docx]

**Supplement 1:**

**CLINCIAN INTERVIEW GUIDE**

*INTERVIEWER INSTRUCTIONS*

1. Ensure that participant survey is completed prior to start of this interview
2. Ensure that verbal consent for interview is completed
3. For WashU interviews – ask ***bold/italicized*** questions first. Interview time cannot exceed 30 minutes.

***INTRODUCTION:***

- Thank you again for agreeing to participate in this study. Your ideas and opinions are very important, and I appreciate you taking the time to share your thoughts with me.
- This interview will be more like a conversation, I have some questions prepared to guide the conversation, but I am very interested in hearing what you would like to share and what you think is important. You are the expert on this topic, and I am here to learn from you.
- We may also contact you to share the results of our interview so that you have the opportunity to provide further input.
- Please keep in mind that there are no ‘right’ or ‘wrong’ answers to any of these questions. If a question is unclear, please ask me to clarify it.
- I will digitally record this interview, as I want to make sure I capture everything you tell me. Your name will not be associated with the recording.
- In your answers, please feel free to tell illustrative stories if it would be helpful. However, we would like to leave out the names of patients, families and your colleagues to protect their privacy.

I will now turn the recording on. Are you ready?

**[TURN ON RECORDING]**

State the date ______________________, time _____________, location _________________, and participant’s unique ID number _________________________.

**Section 1: Typical Workflow**

1. Can we start off with you telling me about the typical workflow you follow when you prescribe antibiotics for a patient 2 years of age or older with acute otitis media (I’ll refer to it as AOM from now on if that’s OK with you)? (Organizational characteristics)

(probe) Tell me about your decision-making process that informs prescribing

**Section 2: Contextual factors that facilitate/impede prescription of short-term antibiotics**

Thanks for sharing that. I would also like to better understand this current practice in general at your clinic and overall organization/agency.

1. ***In general, what are the current practices for treatment of acute otitis media (AOM) at your clinic?*** (Organizational Characteristics)

Probe:

1. What factors influence your decision on when to prescribe antibiotics for children with AOM? (Probe: day of the week, age, organization guidelines, organizational culture resources?)
2. What antibiotics do you usually prescribe for children with AOM?
3. What factors influence your antibiotic choice? (Examples: patient allergy, palatability of medication, side effects of medication, patient age, recurrence, parental desire, length of symptoms)
4. ***How long of an antibiotic duration do you usually prescribe for children 2 years and older with AOM?***
5. ***How do you decide what duration to prescribe? (Examples: habit, clinical practice/it’s what I was taught, local guidelines, national guidelines)***
6. Is the prescribing consistent across clinicians in your practice? (in terms of antibiotic choice and antibiotic duration)
7. Do your local clinics have practice level guidance on prescribing for AOM?
   1. If no, is there any broader system guidance?
   2. If no, would having practice-level guidance be useful for you?
8. Do you have any concerns about overuse of antibiotics and the safety of patients?
9. Have you ever prescribed an antibiotic but told families to wait to fill? Tell me more about how this has worked.
10. We are particularly interested in understanding prescribing of short durations of antibiotics (5-7 days). ***What would facilitate*** ***prescribing shorter durations? Probe: Do you feel this is an important topic? (Organizational perspective)***
    1. ***What “tools”/skills do you use in the event where a patient asks for a longer duration than you plan to prescribe?***
11. ***What difficulties / challenges do you have prescribing shorter durations?*** (Organizational perspective)

**Section 3: Contextual factors that facilitate/impede intervention components**

To facilitate shorter prescribing durations, our research team is designing an intervention. The goal is to increase the number of antibiotic prescriptions for ear infections that use a shorter duration in alignment with national guidelines.

We have 3 primary interventions that I would like to explain to you and get your feedback on. The first intervention will include changes to the **electronic health record** to include links to guidelines and order instructions within antibiotic prescription orders that outline guideline-recommended durations. The second will be **feedback to clinicians on their antibiotic prescribing durations** emailed to them every other month. The third will include **tailored education for parents and clinicians** about guideline-recommended antibiotic durations.

(*Note to interviewer – ask all questions in sections a, b and c. below. Show examples in PowerPoint.)*

***I would like to go through each intervention and get your insight on how you feel about it*** (Implementation and sustainability infrastructure)

1. Our study team will make some **simple changes to the EHR** with a goal of making it easier for clinicians to prescribe guideline-concordant antibiotics. - Show example in PowerPoint (below)
   1. Adding order instructions with recommended dose and duration to amoxicillin, amoxicillin-clavulanate (Augmentin) and cefdinir orderables(prescription ordering fields)
   2. Adding a link to local clinical pathway guidelines to the antibiotic orderables
   3. Adding dosage “quick” buttons
      1. Adding “quick” buttons for 5 (and possibly) 7-day durations


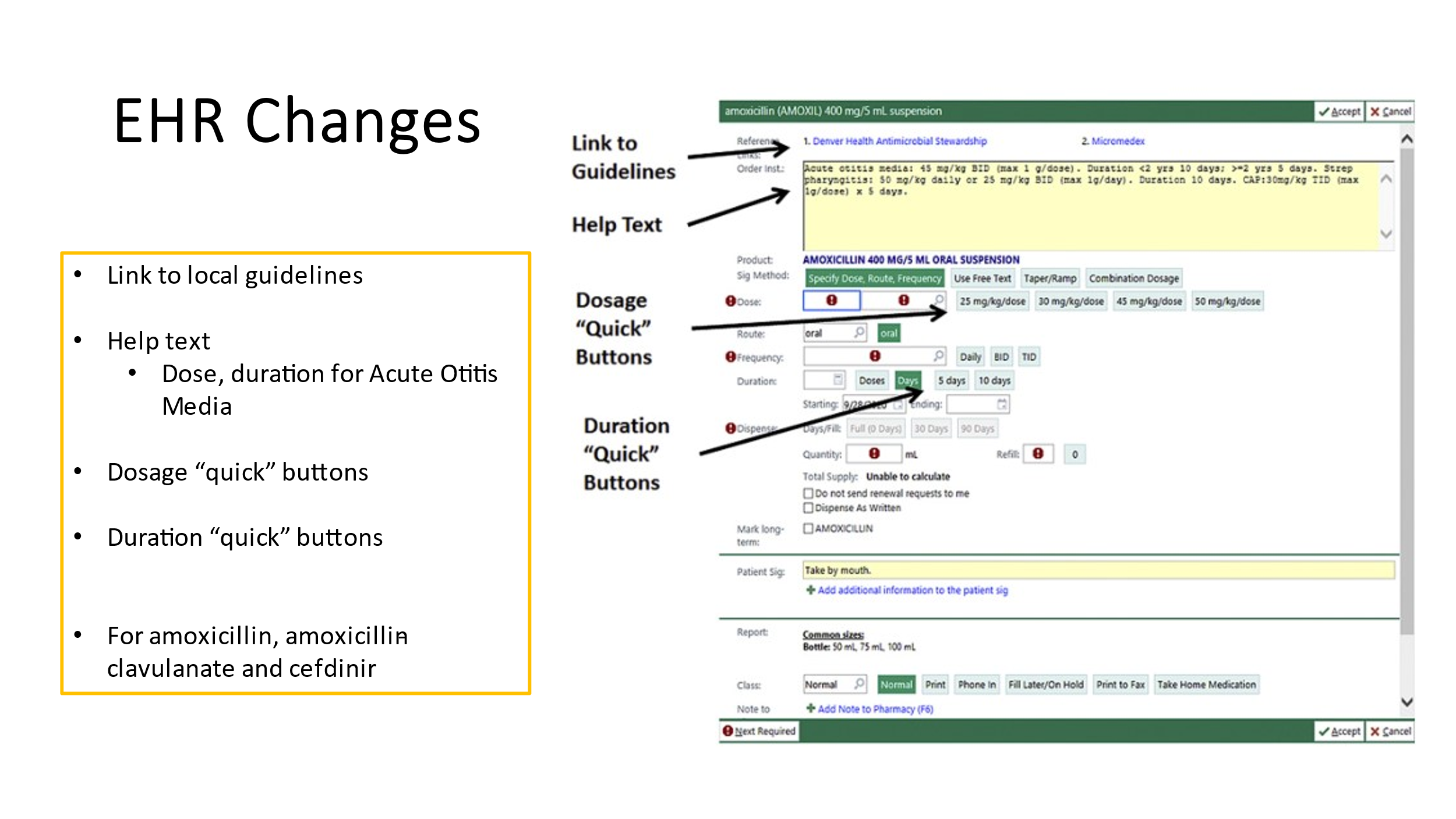


- - 1. What do you like about these EHR changes?
    2. What do you not like about these EHR changes?
    3. How do you think other clinicians will respond?
    4. How will this impact patient care?
    5. What challenges do you envision with using these EHR changes?
    6. What do you think would help to make you use these EHR changes?

1. Does the EHR your organization uses have clinical decision-making tools? Example? Or links to educational resources? Which ones? (Implementation and sustainability infrastructure)
   1. Do you regularly use these tools? If yes why, if no why?
2. How could your EHR help you when making clinical decisions? (Implementation and sustainability infrastructure)
3. **Clinician Feedback** – Show example in PowerPoint (below)
   - 1. Every other month, you would get an email with graphs showing how often you prescribe antibiotics for 5-day durations for children 2 years and older with AOM compared to how your peers are doing.


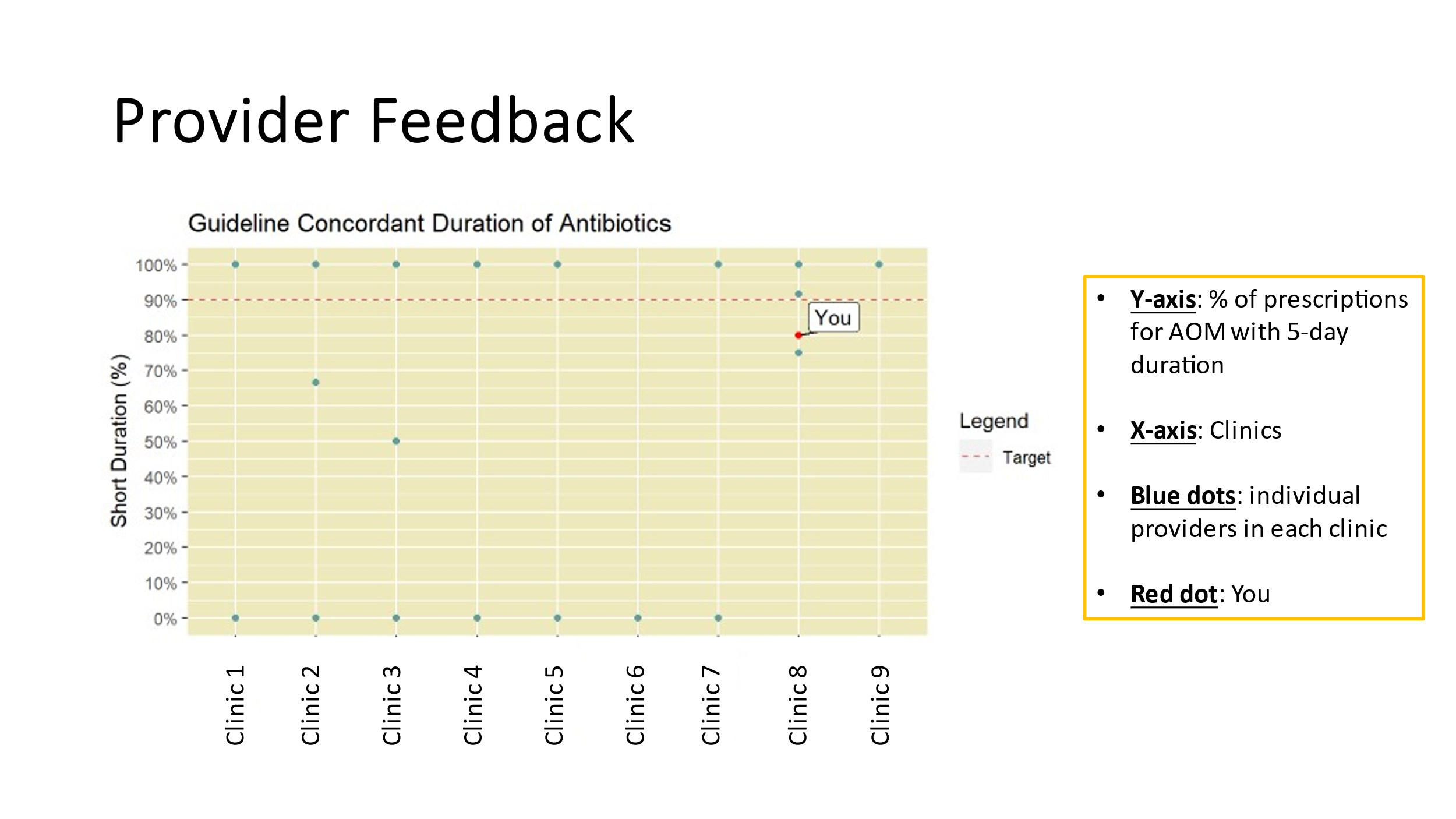


- - 1. What do you like about this feedback?
    2. What do you not like about this feedback?
    3. How do you think other clinicians will respond to this feedback?
    4. How will this feedback impact patient care?
    5. What challenges do you envision with receiving or interpreting this feedback?
    6. What do you think would help to make you pay attention to this feedback?

1. **Education** – Show example in Power Point (below)


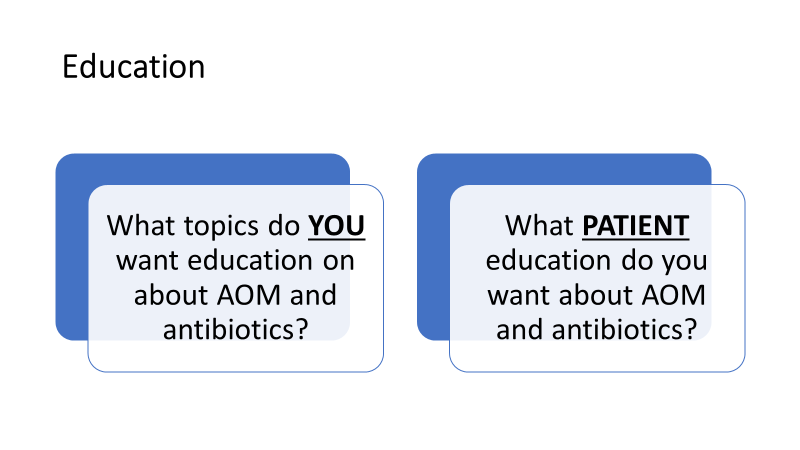


- - 1. As a Clinician, what topics do you want education on about AOM and antibiotics?
    2. What patient education would you want about AOM and antibiotics?

**Overall study**

1. How would participating in this intervention benefit you and other clinicians at your site? (Organizational perspective)
2. How do you think your patients will respond to this project? (Patient perspective)
3. What difficulties or challenges might exist to participating in this project? How would you overcome these barriers? (Organizational perspective)
4. What would make it easier to participate in the intervention? (Implementation and sustainability infrastructure)
   1. What type of incentives would encourage clinicians to participate?
5. How do you typically find out about new information, such as new initiatives? (Organizational characteristics)

**Section 4: Organizational Culture**

1. How do you think your organization’s culture (i.e. general beliefs, values or assumptions that people embrace) will affect the implementation of the interventions? (Implementation and sustainability infrastructure)
2. How / when do clinicians get involved with implementation of new evidence-based guidelines? (Organizational characteristics)
3. Do you currently receive feedback on your prescribing practices from your organization? If so, how are you given this information? (Implementation and sustainability infrastructure)
4. ***To what extent are new ideas embraced and used to make improvements in your organization? Can you give me a recent example? (Organizational characteristics)***
5. Can you describe your working relationship with leaders/ supervisors? (Organizational characteristics)
6. How likely is it that your site would continue or maintain these interventions after the study? What would impact this? (Implementation and sustainability infrastructure)
   1. Explore costs, infrastructure, and clinical, patient and management buy-in

END RECORDING [**TURN OFF RECORDING]**

Thank you so much for sharing your experience on prescribing for AOM. Your contributions will help us to improve our study interventions.

**Administrator INTERVIEW GUIDE**

*INTERVIEWER INSTRUCTIONS*

1. Ensure that participant survey is completed prior to start of this interview
2. Ensure that verbal consent for interview is completed
3. For WashU interviews – ask ***bold/italicized*** questions first. Interview time cannot exceed 30 minutes.

***INTRODUCTION:***

- Thank you again for agreeing to participate in this study. Your ideas and opinions are very important, and I appreciate you taking the time to share your thoughts with me.
- This interview will be more like a conversation, I have some questions prepared to guide the conversation, but I am very interested in hearing what you would like to share and what you think is important. Your clinicians are experts on this topic, and you are an expert in your organization. I am here to learn from you.
- We may also contact you to share the results of our interview so that you have the opportunity to provide further input.
- Please keep in mind that there are no ‘right’ or ‘wrong’ answers to any of these questions. If a question is unclear, please ask me to clarify it.
- I will digitally record this interview, as I want to make sure I capture everything you tell me. Your name will not be associated with the recording.
- In your answers, please feel free to tell illustrative stories if it would be helpful. However, we would like to leave out the names of patients, families and your colleagues to protect their privacy.

I will now turn the recording on. Are you ready?

**[TURN ON RECORDING]**

State the date ______________________, time _____________, location _________________, and participant’s unique ID number _________________________.

We are meeting with clinicians, managers, and administrators to better understand antibiotic prescribing for children 2 years and older with ear infections We are also hoping to learn about current practices for Quality Improvement in your clinic and organization.

**Section 1: Organizational Culture: Workflow for QI**

1. ***How would you describe the culture of your organization when it comes to improving care for children? (general beliefs, values, assumptions that people embrace)? Antibiotic prescribing? Of your own clinic?***
2. ***Can you describe your working relationship with clinicians? (probe*** how do clinicians perceive QI interventions/metrics/data from administrators. Is it a collegial, productive partnership? Adversarial? )
3. ***To what extent are new ideas embraced and used to make improvements in your organization? Can you give me a recent example?***
4. ***How do quality improvement changes get implemented in your organization?***
5. ***How / when do your clinic/clinicians get involved with implementation of new quality improvement measures? What does their involvement look like?***
6. Antibiotic specific: In thinking about antibiotic prescribing,

Do clinicians receive data on their performance for QI metrics from your organization? Does this include data on antibiotic prescribing practices?

If so,

- what information and how are they given this information?
- Have there been any changes to antibiotic use as a result of these quality metrics?

1. Do you know of any clinicians championing efforts to improve care for children with ear infections or to reduce the number of antibiotics prescribed to children? What is your clinic’s perspective on this? What is your perspective?
2. How do you and your clinicians typically find out about new information, such as new clinical initiatives? Or new eHR helpful tools/ improvements? Are these easy or hard to implement?

**Section 2: Contextual factors that facilitate/impede prescription of shorter lengths of antibiotics (e.g. 5 instead of 10 days)**

1. To what extent are new ideas embraced and used to make improvements in your clinic and by the clinicians? Can you provide a recent example?
2. What difficulties/ challenges might exist to clinicians or clinics recommending/prescribing shorter durations of antibiotics?

**Section 3: Contextual factors that facilitate/impeded intervention components**

To increase the use of shorter (instead of longer) antibiotic durations, our team is designing a quality improvement intervention. The goal is to increase the number of prescriptions for ear infections that use the latest guidelines for antibiotic durations.

We have 3 primary interventions that I would like to explain to you and get your feedback on. (show them PowerPoint slides for each intervention as describe)

The **first** intervention will include changes to the **electronic medical record** to make prescribing easier and faster for clinicians.

The **second** will be given to clinicians in some clinics but not to others and includes providing feedback on their antibiotic prescribing durations emailed to them every other month.

The **third** will include tailored education for parents and clinicians about guideline-recommended antibiotic durations.


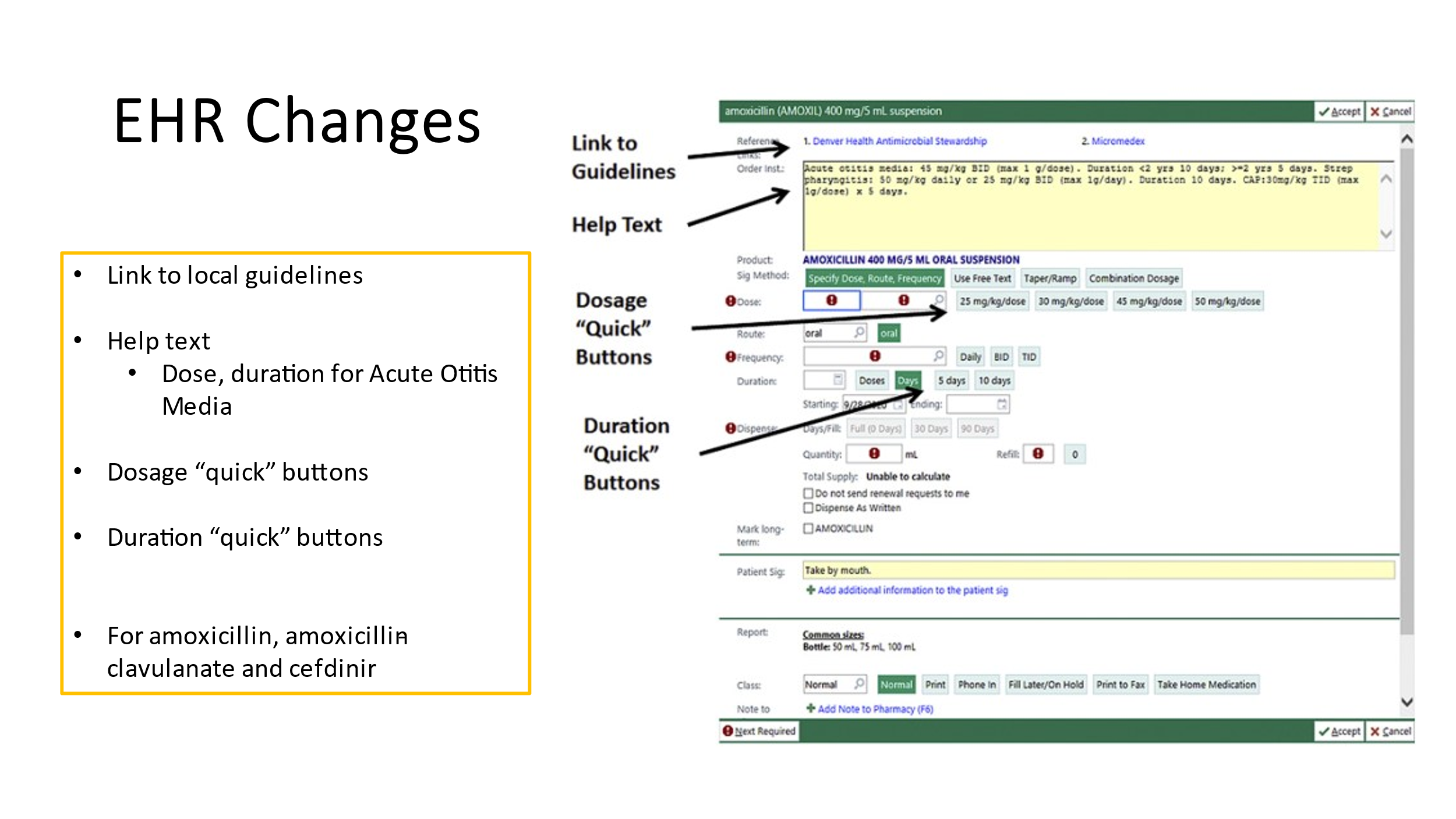


***
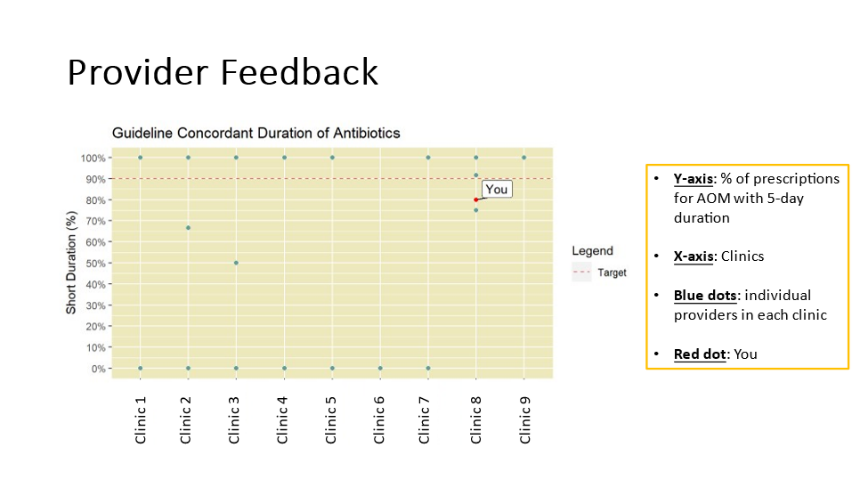
***

***
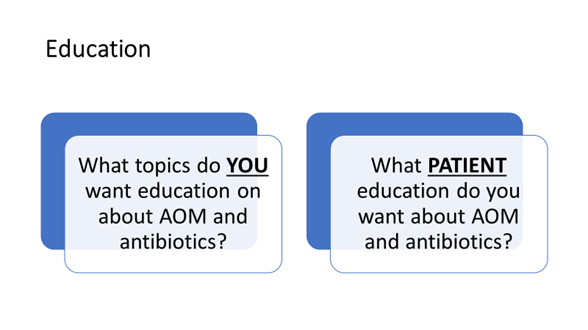
***

1. ***What are your thoughts on the benefits to your clinic for participating in this intervention?***
2. ***Can you tell me about challenges or difficulties that would hinder participation at your organization or clinic? Do you have suggestions for overcoming these barriers?***
3. How well does the intervention fit with existing work processes and practices in your setting? What are likely issues or complications that may arise?
4. How do you think your clinicians will respond?
5. What recommendations do you have for rolling out the interventions– communicating with clinicians?
6. ***Tell me about facilitators to implementing and maintaining the intervention at your facility? What can make it easy to implement and maintain?***
7. ***What kinds of infrastructure changes will be needed to accommodate the intervention? Changes in scope of practice? Changes in formal policies? Changes in information systems or electronic records systems?***
8. ***Is there anyone else we haven’t thought of who would need to be involved?***
9. ***What kind of approvals will be needed? Who will need to be involved? Can you describe the process that will be needed to make these changes?***
10. ***Are you concerned about the time it will take to educate and implement the EHR changes? Or that it will add time to visits? If so, what questions or concerns do you have that we need to keep in mind?***
11. ***What educational topics do you think are needed for clinicians and for parents?***

*PATIENT PERSPECTIVE*

I would like to talk a little bit now about how you obtain patient input into QI efforts like this.

1. ***How does the clinic obtain patient feedback? When you implement QI interventions?***
2. ***How do you think the interventions will meet the needs of the patients served by your organization?***
3. ***How do you think patients served by your org will respond to the intervention and the shorter prescribing guidelines for ear infections?***
4. ***What patient issues do you foresee (like parents calling to ask for scripts of antibiotics if the clinician used “watch and wait” or calling and wanting a long course of antibiotics sent in).***

*EXTERNAL ENVIRONMENT*

Cosmopolitanism (CFIR Construct)

1. Do you network with colleagues or people in similar professions/positions outside your setting? What are the venues - Listservs? Local or national conferences? Trainings?
2. Do you discuss clinical quality improvement initiatives or EHR improvements? or more generally about your profession?
3. To what extent does your organization encourage its clinicians to network with colleagues outside your own setting? Are they able to attend local/national conferences? Other venues?

END RECORDING [**TURN OFF RECORDING]**

Thank you so much for sharing your experience on prescribing for ear infections. Your contributions will help us to improve our study interventions.

**Focus Groups with Parents – Guide**

*When participants log onto online platform, welcome them. Tell them the discussion will begin shortly.*

1. **Welcome**

Good Morning/Afternoon. Thank you for joining us today. My name is __________, and my role today is to guide our discussion and to encourage everyone to share their thoughts and ideas. I’d also like to introduce [name of research assistant] who will be taking notes during group discussion today and monitoring the chat.

The overall goal of our project is to figure out the best way to get clinicians to prescribe short courses of antibiotics for ear infections for children 2 years of age and older. We have already conducted interviews with clinicians to get their thoughts on care and on prescribing antibiotics for children with ear infections. Today, we have a group format to talk more about this topic with parents.

1. **Focus Group Procedures and Logistics**

Before we begin, let me mention a few things about how we usually conduct these groups:

1. I am the group facilitator. My role is to ask the questions we have for the group and to encourage everyone to participate. I won’t be doing much talking, but may ask you to explain more or to give an example. Also, it’s my job to see that everyone has a chance to voice their opinions, as well as to keep us moving along so that we have time to discuss all the questions. If it seems as though I am cutting you off, this is NOT meant to be rude. I will just be trying to make sure that we have enough time to have a complete discussion of each question. This group should last about an hour.
2. It’s really important that everyone hear this: THERE ARE NO RIGHT OR WRONG ANSWERS!!! Each person’s experiences and opinions are valid, and we want to hear a wide range of opinions on the questions we’ll be asking. So, please speak up, whether you agree or disagree with what’s being said, and let us know what you think.
3. Participation in this group is completely voluntary. **Because you are participating in a group discussion, it is possible that what you say may not be kept confidential.** We strongly encourage everyone in this group to ensure that what is said here is not shared with others outside of the online “room.”
4. Let me tell you about our recording process. We are using an online platform to record our discussion. We need to record these focus groups because we want to capture everything that all of you say, and we simply can’t write fast enough to get it all down. We’ll be using only first names in the group discussion, and when we put together the results from all the groups, we won’t include any names at all.
5. Since this is an online platform, there are two ways for you to join the conversation. You can either unmute yourself and ask a question or you can use the chat box and chat directly with me, or the entire group. Let me show you how each of these work.
6. If there is any feedback that you would prefer not to share out loud, that you prefer not to be recorded, or that you didn’t have a chance to say, please put it in the chat box directly to me or the whole group.

Does anyone have any questions at this point on the group or what is expected of you?

[MODERATOR PAUSES TO ANSWER ANY QUESTIONS]

[Activate record function]

To get us started, I will call out each person and in about 2 minutes, if you can tell us

- Your first name, your children and their ages, and anything you would like to share about your experiences with ear infections

You each shared an experience with ear infections (could summarize if needed) I would like to talk a little more about these experiences.

1. How do you decide when to take your child (2 years of age or older) to see a clinician for an ear infection?
   1. What are your expectations when you see your clinician?
   2. What are your expectations around medication/prescriptions?
2. Let’s talk specifically about a time when your child/children were diagnosed with an ear infection?
   1. Tell me about what treatment was offered/recommended?
      1. (probe) what is/was your treatment preference?
   2. Was your child prescribed antibiotics? If so:
      1. For how long?
      2. What were you told about the medication?
      3. were methods other than antibiotics discussed?
      4. Did your child take the antibiotic for the entire length prescribed?
   3. Has your child ever been diagnosed with an ear infection, but an antibiotic was not prescribed? Tell me how that made you feel and what happened.
   4. Did your clinician talk with you about the risks and benefits of using an antibiotic compared to not using one?
   5. How important is it to you to have a method of pain control, like Ibuprofen (Motrin/Advil) or Acetaminophen (Tylenol), included in the treatment plan for your child’s ear infection?
3. Do you know how your clinician decides what antibiotic to prescribe, and for how long? (Typical antibiotic lengths are i.e. 5 days, 7 days, 10 days)
4. What length of days would you prefer for treating an ear infection? Why?
   1. How would you feel if your child was prescribed the shortest antibiotic length?
5. If your clinician provides a prescription for an antibiotic and asks that it **only** be taken if your child feels worse or doesn’t improve within 72 hours, what would you do?
   1. Probe (if not answered): Would you fill the prescription right away, wait, or take another course of action?
   2. Would you wait to give to your child? How long?
   3. What would make you decide to give your child the medication?
   4. What might help you feel more comfortable with this method of treatment?
6. What concerns do you have if your child’s ear infection is treated with an antibiotic?
   1. Are you concerned about side effects from antibiotics? Which ones?
   2. Has your child ever experienced any side effects from antibiotics prescribed for ear infections? Examples?
   3. Have you ever experienced challenges when getting antibiotics for your child?
7. What are your concerns if your child’s ear infection is not treated with an antibiotic?
8. What is the best way to inform parents/caregivers about treatment for ear infections in children?

- An example of information we think the general public may not know
  - 85% of children will recover from ear infections without an antibiotic.
    - Do you think most parents know this?
    - Would they agree with this or feel comfortable letting children recover with pain control, but without an antibiotic?
  - When ear infections are treated, in children ages 2 and older can be treated with just a 5-day course of antibiotics
    - Do you think most parents know this?
    - Would they agree with this or feel comfortable with a short duration of antibiotics?

Probe: How to give this information to parents? What other information might they need?

1. Thanks again for your time and your feedback. Is there anything else you think we should know about treating ear infections and reducing antibiotic use among children? Also feel free to put anything in the chat as we will copy all of that information.
