# Supplementary material for: Reducing antibiotic duration for acute otitis media: clinician, administrator, and parental insights to inform implementation of system-level interventions
Source: Antimicrob Steward Healthc Epidemiol. 2025 Jan 6;5(1):e3. doi: 10.1017/ash.2024.469 (PMC11704944; doi:10.1017/ash.2024.469)
Supplement: Rinehart et al. supplementary material 2 — Rinehart et al. supplementary material [file S2732494X24004698sup002.docx]

**Supplement 2:**

*Data Analysis*

After completing each interview, the interviewing researcher listened to the audio recording and created a two-page summary for each critical domain. A second team member (clinician) listened to the recording, confirmed, and added data to the original summary document. The study team consolidated themes and findings from the summaries into a single interview matrix representing the key domains (columns) for each interview (rows). In addition, four columns documented the following for each interview: high-level summary, unique findings, illustrative quotes, and intervention implications. Data were further reduced by creating a thematic matrix summarizing themes by key domains. Once completed, this visual representation of the data allowed the team to identify connections and reach a consensus on emergent themes and key findings across all interviews. The research team met weekly to identify emergent themes within the key domains, topics needing further exploration, and topic saturation. The research team also met regularly with the broader project study team, inclusive of leadership and clinical staff across the three sites, to obtain feedback on preliminary findings.
